# Tumor Enucleation for Renal Cell Carcinoma

**DOI:** 10.15586/jkcvhl.2015.27

**Published:** 2015-04-04

**Authors:** Zachary L. Smith, S. Bruce Malkowicz

**Affiliations:** Division of Urology, Hospital of the University of Pennsylvania, Philadelphia, Pennsylvania, USA

## Abstract

The increased number of small renal masses (SRMs) detected annually has led to a rise in the use of nephron-sparing surgery (NSS). These techniques aim to preserve the largest amount of healthy renal tissue possible while maintaining the same oncologic outcomes as radical nephrectomy (RN). Additionally, partial nephrectomy (PN) has been linked to a lower risk of chronic kidney disease, cardiovascular morbidity, and mortality when compared to RN. There has been continual progress toward resecting less renal parenchyma. While the predominant surgical method of performing NSS is through traditional PN, simple enucleation (SE) of the tumor has increased in popularity over recent years. SE is a technique that aims to preserve the maximal amount of renal parenchyma possible by utilizing the renal tumor pseudocapsule to bluntly separate the lesion from its underlying parenchyma, offering the smallest possible margin of excised healthy renal tissue. Several studies have demonstrated the oncological safety of SE compared with PN in the treatment of SRMs, with lower overall incidence of positive surgical margins. Additionally, SE has been shown to have similar 5- and 10-year progression-free and cancer-specific survival as PN. We present a review of the literature and an argument for SE to be a routine consideration in the treatment of all renal tumors amenable to NSS.

## Introduction

It is estimated that there will be 61,560 new cases of kidney cancer in the United States in 2015, and an estimated 14,080 deaths (1). This incidence has been on the rise over the last three decades and is generally attributed to the increased utilization of cross-sectional imaging across all disciplines (2,3). Despite this increasing incidence, the estimated 5-year relative survival rate has improved from 50% in 1975–1977 to 74% in 2004–2010 (1). This improvement in survival may be ascribed to the stage migration which has been seen over the last two decades, with more patients presenting at stage I than any other stage (4). Alternatively, it could be attributed to the paralleling increase in treatment of renal tumors with improved surgical techniques and medical therapies (3).

## Nephron-sparing surgery

This increased number of low stage small renal masses (SRMs) detected annually has led to an evolution in the treatment of renal cell carcinoma (RCC); specifically, a rise in the use of nephron-sparing surgery (NSS) (4–7). These techniques aim to preserve the largest amount of healthy renal tissue possible while maintaining the same oncologic outcomes as radical nephrectomy (RN). Additionally, partial nephrectomy (PN) has been linked to a lower risk of chronic kidney disease (CKD), cardiovascular morbidity, and mortality when compared to RN (8–11).

While the use of NSS can be traced as far back as the late 19th century (12), the importance of preventing development or worsening of CKD has become increasingly evident over recent years. It is thought that resultant CKD is likely the root of the cardiovascular and all-cause mortality seen in patients treated with RN or PN (10,13,14). Unfortunately, one study found a baseline CKD (stage III or greater) in 22% of patients presenting for surgical management of their renal tumors, with this incidence increasing to 40% in patients aged 70 years (15). For these reasons, current guidelines support the use of NSS for the treatment of the SRM whenever technically feasible (6,16).

## Simple enucleation

In 1950, Benjamin Abeshouse wrote “Few procedures provide the urologist with more satisfaction than those that preserve renal function” (17). While Dr. Abeshouse may have practiced urology prior to the availability of the robust data we now possess, his statement rings true to this day. On this principle, NSS has taken a prominent position at the helm of the treatment of renal tumors. Likewise, there has been continual progress toward resecting less and less renal parenchyma. While the predominant surgical method of performing NSS is through traditional PN, simple enucleation (SE) of the tumor has increased in popularity over recent years (18–21). SE is a technique that aims to preserve the maximal amount of renal parenchyma possible by utilizing the renal tumor pseudocapsule to bluntly separate the lesion from its underlying parenchyma. This method of NSS has been used for more than three decades with success (22–24). The largest contribution to the body of literature on SE has been published by the University of Florence group and their affiliates (18,19,21,25–30).

### Technique

The renal parenchyma adjacent to the tumor is incised. Using a blunt dissecting instrument (e.g. empty knife handle, closed Metzenbaum scissors, small Yankauer suction tip), the tumor and its pseudocapsule are bluntly separated from the adjacent renal parenchyma. This natural cleavage plane between the tumor and the normal parenchyma allows for removal of the lesion without concomitant removal of any visible rim of normal renal tissue. Any large vessels traversing this plane can be ligated with clips or sutures during the removal. As with PN, following removal of the lesion, the resection site may be ablated with an energy source (e.g. Nd-YAG laser or Argon beam laser).

The procedure may be performed with or without hilar vessel clamping. Unlike PN, SE is often met with much less bleeding when done without vessel clamping due to the lack of any sizable entrance into renal parenchyma (19,20). Additionally, the procedure may be performed in an open or robotic-assisted laparoscopic fashion with equivalent outcomes (19,25). These authors have published their own institutional experience and methods previously (20).

## Surgical margins

While traditional thinking was that a 1 cm margin was required during PN, this has been challenged and disproven in recent years. Many studies have now supported margins of all sizes—including <1 mm—as being safe, noting that there is no minimal requirement to maintain an oncologically sound resection (31–36). These principles have been supported in masses up to 7 cm (37). Given these results, the European Association of Urology recommends obtaining the minimal tumor-free surgical margin of healthy tissue that is required, thus reducing the risk for local recurrence while minimizing any detriment to renal function (6).

Overall, positive surgical margins (PSMs) are relatively rare events at the time of NSS, with current literature identifying positive surgical margin (PSM) rates after PN to range from 0% to 7% (29,38–41). When investigating the significance of a PSM on final pathological analysis, little effect on survival has been shown (40–42). While a large international, multi-institutional study found that PSMs may be associated with an increased risk of recurrence (10.1% vs 2.2%, p=<0.0001), they found no effect on overall, cancer-specific, or recurrence-free survival (42).

Minervini et al have published multiple times on the role of pseudocapsule penetration on rates of PSM and oncological outcomes (26,27). In their most recent analysis of patients undergoing SE, 51% of specimens had an intact pseudocapsule free from neoplastic invasion, 35% had capsular penetration on the parenchymal side, and 14% had invasion into the perirenal adipose tissue (left attached to surface of tumor). None of the patients had PSMs on final analysis and the 5-year progression-free survival (PFS) rates were the same for the first two groups, and only worsened with perirenal adipose tissue invasion (27). Additionally, penetration into and beyond the pseudocapsule is accompanied by a thin layer of parenchymal tissue even when no efforts are made to leave a rim of healthy kidney tissue around the neoplasm (26). Thus, with or without microscopic invasion into the pseudocapsule, patients undergoing SE tend to maintain good surgical margin and survival rates.

Several studies have demonstrated the oncological safety of SE compared with PN in the treatment of SRMs, with lower overall incidence of PSMs (28–30). In a large, multicenter retrospective series, the Surveillance and Treatment Update Renal Cancer (SATURN) study found a PSM rate of 0.2% with SE and 3.4% with PN (p=<0.001) (28). Similarly, the prospective Italian Registry of Conservative Renal Surgery (RECORd) project found PSM rates of 1.6% and 7.4% with SE and PN, respectively (p=<0.001) (29).

## Survival outcomes

Long-term oncologic equivalence between SE and PN is now well established (20,28,30,43). Carini et al (30) published their experience of SE for SRMs (<4 cm) in 232 patients with a mean follow up of 76 months, demonstrating 5- and 10-year cancer-specific survival (CSS) of 97% and 95%, respectively. Five- and 10-year PFS was 96% and 94%, respectively, and there were no PSMs or local recurrences. Recurrence rates for tumors up to 7 cm in the same series also demonstrated similar CSS and PFS rates to RN and PN.

The SATURN study published long-term oncological outcomes comparing SE (537 patients) to PN (982 patients) at 54 month and 51 month mean follow-up, respectively (28). Similar to the above study, this multicenter review also found no difference in PFS or CSS between techniques. The 5- and 10-year PFS were 91% and 91% after SE and 89% and 82% after PN, respectively (p=0.09). The 5- and 10-year CSS were 94% and 93% after SE and 94% and 92% after PN, respectively (p=0.94).

While most SE studies have focused predominantly on comparison to PN, it has also been compared against RN and shown to have similar outcomes. Minervini et al compared 332 patients who underwent SE to 143 matched patients who underwent RN with a mean follow-up of 72 and 58 months, respectively (21). They found 5-year and 10-year PFS rates of 95% and 93% for SE and 91% and 89% for RN, respectively (p=“non-significant”). Five- and 10-year CSS rates were 94% and 94% for SE and 92% and 89% for RN, respectively (p=“non-significant”).

## Conclusions

Contemporary literature has shown SE to be a widely accepted technique of NSS. There is an abundance of data to support SE as an oncologically sound alternative to PN in appropriately selected tumors. In the arena of renal parenchyma preservation, SE offers the smallest possible margin of excised healthy renal tissue. SE should be a routine consideration for the treatment of all renal tumors.
